# The impact of slow stomatal kinetics on photosynthesis and water use efficiency under fluctuating light

**DOI:** 10.1093/plphys/kiab114

**Published:** 2021-03-08

**Authors:** David Eyland, Jelle van Wesemael, Tracy Lawson, Sebastien Carpentier

**Affiliations:** 1 Division of Crop Biotechnics, Laboratory of Tropical Crop Improvement, KU Leuven, Leuven, Belgium; 2 School of Life Sciences, University of Essex, Colchester, Essex, UK; 3 Bioversity International, Banana Genetic Resources, Leuven, Belgium

## Abstract

Dynamic light conditions require continuous adjustments of stomatal aperture. The kinetics of stomatal conductance (*g_s_*) is hypothesized to be key to plant productivity and water use efficiency (WUE). Using step-changes in light intensity, we studied the diversity of light-induced *g_s_* kinetics in relation to stomatal anatomy in five banana genotypes (*Musa* spp.) and modeled the impact of both diffusional and biochemical limitations on photosynthesis (*A*). The dominant *A* limiting factor was the diffusional limitation associated with *g_s_* kinetics. All genotypes exhibited a strong limitation of *A* by *g_s_*, indicating a priority for water saving. Moreover, significant genotypic differences in *g_s_* kinetics and *g_s_* limitations of *A* were observed. For two contrasting genotypes, the impact of differential *g_s_* kinetics was further investigated under realistic diurnally fluctuating light conditions and at the whole-plant level. Genotype-specific stomatal kinetics observed at the leaf level was corroborated at whole-plant level by transpiration dynamics, validating that genotype-specific responses are still maintained despite differences in *g_s_* control at different locations in the leaf and across leaves. However, under diurnally fluctuating light conditions the impact of *g_s_* speediness on *A* and intrinsic (_i_WUE) depended on time of day. During the afternoon there was a setback in kinetics: absolute *g_s_* and *g_s_* responses to light were damped, strongly limiting *A* and impacting diurnal _i_WUE. We conclude the impact of differential *g_s_* kinetics depended on target light intensity, magnitude of change, *g_s_* prior to the change in light intensity, and particularly time of day.

## Teaser

Whole-plant transpiration rapidity corroborated leaf-level stomatal kinetics in banana, but impacts of stomatal dynamics under fluctuating light depended on other factors like time of day.

## Introduction

In order to survive, plants need to balance CO_2_ uptake for photosynthesis (*A*) with water loss via transpiration. By adjusting their aperture, stomata control gaseous exchange between the leaf interior, and the external atmosphere. Stomatal aperture is adjusted by moving solutes into or out of the guard cells. These changes in osmotic potential elicit water movement in or out of the guard cells, altering turgor pressure and subsequently aperture. In general, stomatal opening in well-watered C3 and C4 species is triggered by high light intensity, low vapor pressure deficit (VPD), and low CO_2_ concentrations. Opposite environmental conditions (low light, high VPD, and high CO_2_) stimulate stomatal closure (Assmann and Shimazaki, 1999; Outlaw, 2003; Lawson and Morison, 2004). Therefore, in a dynamic field environment, stomata are continuously adjusting the aperture to achieve an appropriate balance between carbon gain and water loss (Pearcy, 1990; Lawson and Blatt, 2014). Most research has studied stomatal conductance (*g_s_*) and *A* under steady-state conditions. A high *g_s_* under steady-state conditions is associated with high *A* and consequently improved growth (Fischer et al., 1998; Franks, 2006). However, as *g_s_* kinetics are typically a magnitude slower than those of *A*, the speed at which these steady-state values are reached in a fluctuating environment have a great influence on the growth and water use efficiency (WUE; Lawson and Blatt, 2014; Kaiser et al., 2016; McAusland et al., 2016; Taylor and Long, 2017; De Souza et al., 2020; Yamori et al., 2020). In a fluctuating field environment, light intensity is one of the most variable environmental conditions as it changes continuously by moving cloud covers and shading from adjacent plants (Pearcy, 1990; Slattery et al., 2018; Morales and Kaiser, 2020). In this way, stomata frequently experience alternating light intensities, inducing stomatal responses that change *A*, *g_s_*, and the ratio of these, the intrinsic WUE (_i_WUE). The balance between CO_2_ gain and H_2_O loss under changing light intensities is disturbed by delayed *g_s_* responses (Vialet-Chabrand et al., 2017; Slattery et al., 2018). Limitations of *A* after an increase in light intensity are the combination of diffusional and biochemical limitations. Biochemical activation has been shown to majorly limit *A* during short light flecks (Soleh et al., 2017; Taylor and Long, 2017; Acevedo-Siaca et al., 2020). Under longer light periods, limitations have been mainly attributed to stomatal limitations, with biochemical activation only limiting for a short time (<10 min) because of rapid activation of RuBP regeneration and Rubisco (Mott and Woodrow, 2000; Kaiser et al., 2016; Deans et al., 2019a; De Souza et al., 2020). The slower *g_s_* increase to increased light intensity limits the CO_2_ uptake for *A*, while the slower *g_s_* decrease to decreased light intensity results in unnecessary water loss. The limitation of *A* by the slower kinetics of *g_s_* has been shown to be significant in well-watered C3 species (Farquhar and Sharkey, 1982; Jones, 1998; Lawson and Blatt, 2014; McAusland et al., 2016; Deans et al., 2019a). Rapid *g_s_* kinetics, therefore, have been hypothesized to maximize *A* and _i_WUE, as steady-state values under the new conditions can be rapidly achieved (Lawson and Blatt, 2014; Papanatsiou et al., 2019; De Souza et al., 2020; Kimura et al., 2020). The *g_s_* kinetics are, together with the final steady-state *g_s_* the plant reaches, crucial to determine the plant performance (Franks and Farquhar, 2007; Vico et al., 2011; McAusland et al., 2016; Qu et al., 2016; Faralli et al., 2019b; Yamori et al., 2020). The importance of diversity in *g_s_* kinetics was highlighted by De Souza et al. (2020), who showed a three-fold higher variability in carbon assimilation between cassava genotypes under fluctuating light than under steady-state conditions, mainly caused by differences in stomatal limitation. However, to our knowledge, the diversity of *g_s_* kinetics across varieties has neither been investigated at whole-plant level nor under diurnally fluctuating light conditions.

Here our research aimed to explore biodiversity in light-induced stomatal dynamics across genotypes and evaluate for the first time the impact on whole-plant level. We studied the diversity of light-induced *g_s_* kinetics in relation to stomatal anatomy in five banana genotypes (*Musa* spp.) with distinct transpiration phenotypes (van Wesemael et al., 2019). We modeled the impact of diffusional and biochemical kinetics on *A* under single step-changes in light intensity and modeled the impact of differential *g_s_* kinetics on *A* and _i_WUE under realistic diurnal fluctuating light conditions. By comparing the *g_s_* kinetics in response to step-changes with the *g_s_* responses under fluctuating light conditions, we gain insight into the importance of stomatal kinetics on diurnal carbon gain and WUE.

## Results

### *A* and *g_s_* response to step changes

**Figure 1:**
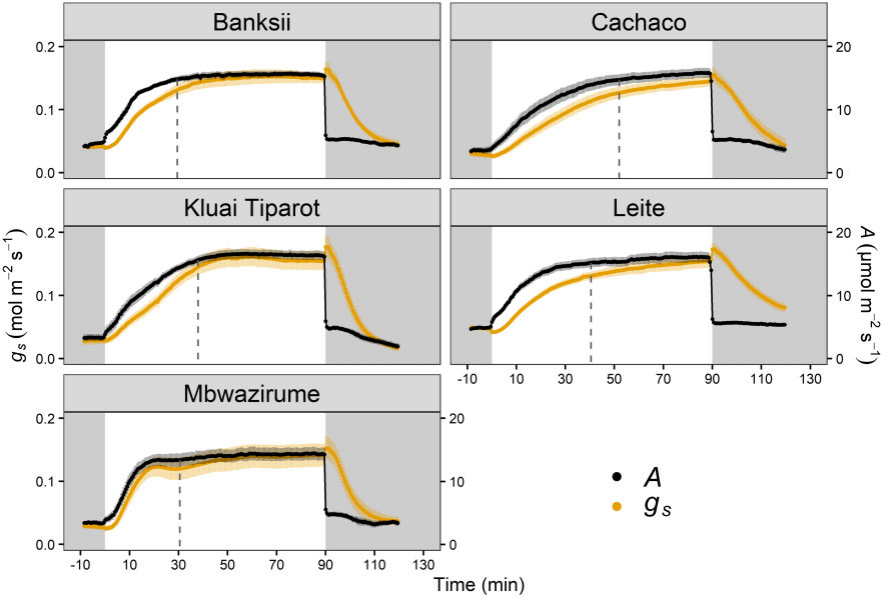
Response of *g_s_* (orange) and *A* (black) of five banana genotypes to a step increase in light intensity from 100 to 1,000 µmol m^−2^s^−1^ followed by a decrease from 1,000 to 100 µmol m^−2^s^−1^. Grey and white areas indicate time periods of 100 µmol m^−2^ s^−1^ and 1,000 µmol m^−2^s^−1^, respectively. Dashed lines indicate when 95% of steady-state *A* was reached. Points and error bars represent mean ± SE (*n* = 7–8).

Increasing light intensity from 100 to 1,000 µmol m^−2^ s^−1^ induced a strong stomatal opening response (Figure 1). The *g_s_* response followed a sigmoidal pattern. A similar sigmoidal limiting pattern was observed for *A* in all genotypes, indicating a strong limitation of *A* by *g_s_* in banana (Figure 1). Between genotypes, there were significant differences in the speed of *g_s_* increase. Steady-state *A* and *g_s_* under high light intensity were reached in three out of five genotypes. In contrast, the genotypes Cachaco and Leite continued to increase *g_s_* and *A* slowly after 90 min of 1,000 µmol m^−2^ s^−1^. The subsequent decrease in light intensity from 1,000 to 100 µmol m^−2^ s^−1^ resulted in a rapid *g_s_* decrease, which also followed a sigmoidal pattern (Figure 1). Photosynthesis, on the other hand, as expected decreased instantly because light became the limiting factor (Figure 1).

### Modeling steady-state and light-induced responses of *g_s_*

**Figure 2:**
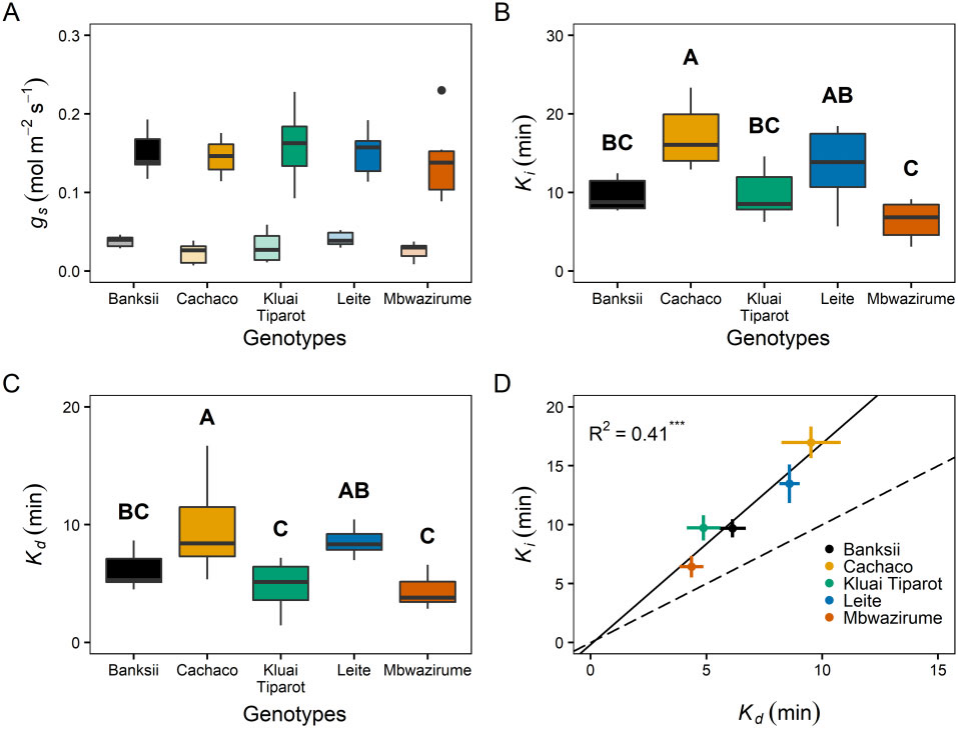
Modeled steady-state and light-induced variables of the *g_s_* response to a step increase and decrease in light intensity between 100 and 1,000 µmol m^−2^ s^−1^ for five different banana genotypes (*n* = 7–8). A, Steady-state *g_s_* at 100 (*g_s_*_,100_ faded colors) and 1,000 µmol m^−2^ s^−1^ (*g_s_*_,1,000_ bold colors). B, Time constant of *g_s_* increase (*K_i_*) for different genotypes. Different letters indicate significant differences between genotypes (post hoc Tukey HSD test, *P* < 0.05; A > B > C). C, Time constant of *g_s_* conductance decrease (*K_d_*) for different genotypes. Different letters indicate significant differences between genotypes (post hoc Tukey HSD test, *P* < 0.05; A > B > C). D, Significant correlation between *K_i_* and *K_d_* (Pearson’s correlation, *R*^2^ = 0.41, *P* < 0.001). *K_i_* was significantly higher than *K_d_*. The solid line shows the linear regression, the dashed line shows the 1:1 line. Points and error bars represent mean ± se (*n* = 7–8). The bold middle line in boxplots represents the median. The box is confined by the first and third quartile and the whiskers extend to 1.5 times the interquartile distance. Points falling outside the whiskers are considered outliers and plotted as dots.

The steady-state *g_s_* at 100 µmol m^−2^ s^−1^ (*g_s_*_,100_) and 1,000 µmol m^−2^ s^−1^ (*g_s_*_,1,000_) did not differ significantly between genotypes (Figure 2A;Supplemental Table S1). *g_s_*_,100_ ranged from 0.023 to 0.040 mol m^−2^s^−1^, while *g_s_*_,1,000_ ranged between 0.14 and 0.16 mol m^−2^s^−1^ (Figure 2A;Supplemental Table S1).

The speed of *g_s_* increase varied strongly between the banana genotypes and the modeled variables differed significantly (Figure 2, B and C; Supplemental Table S1). The genotype with the slowest *g_s_* increase, Cachaco, had an average time constant *K*_i_ of 17 min, while the fastest genotype, Mbwazirume, had a *K*_i_ of 6.4 min (Figure 2B;Supplemental Table S1). The speed of the decrease in *g_s_* (*K*_d_) was also genotype-dependent (Figure 2C;Supplemental Table S1). *K*_d_ was about two-fold higher in Cachaco (9.5 min) than in Mbwazirume (4.4 min). Across all genotypes, *K*_i_ was significantly correlated with *K*_d_ (*R*^2^ = 0.41, *P* < 0.001; Figure 2D;Supplemental Figure S1). However, the decrease in *g_s_* was significantly faster than the increase (*P* <0.001). *K*_i_ was significantly correlated with the time to reach 95%, 90%, and 50% of steady-state *g_s_* under the high light intensity (*R*^2^ = 0.27–0.57, *P* < 0.001; Supplemental Figure S1). Also the maximal slope of *g_s_* increase and decrease (*Sl_max_*_,i_ and *Sl_max_*_,d_) were significantly correlated with the time constant *K* as the magnitude of *g_s_* change was similar across genotypes (*R*^2^ = 0.52 and 0.49 for *g_s_* increase and decrease, respectively, *P* < 0.001; Supplemental Figure S1). During light-induced stomatal opening comparable differences across genotypes were present in *Sl*_max,i_ as in *K*_i_. The lowest *Sl*_max,i_ values were observed for the genotype Cachaco and the highest values for Mbwazirume (Supplemental Figure S2 and Supplemental Table S1). *Sl*_max,d_ was highest for the genotype Kluai Tiparot, while Leite showed the lowest *Sl*_max,d_ (Supplemental Figure S2 and Supplemental Table S1). Analogous to the opening and closing time constant, the absolute slope of closing was significantly higher than the opening slope (*P* < 0.001).

### Impact of stomatal opening speed on *A*

**Figure 3:**
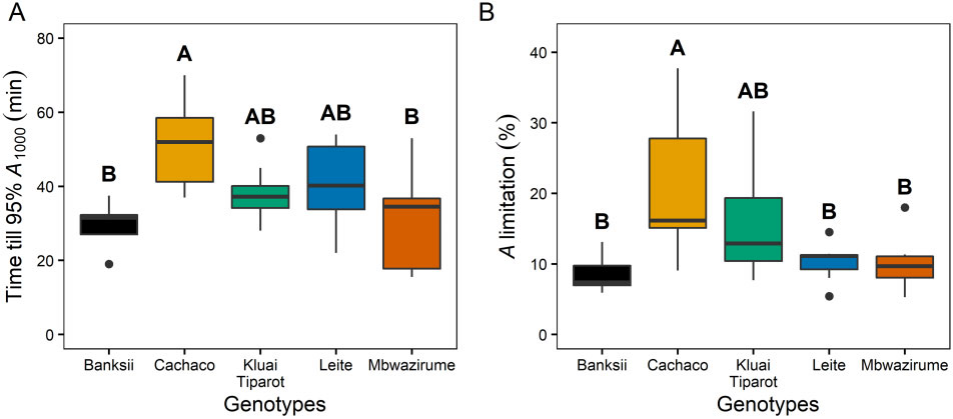
Limitation of *A* after the increase in light intensity from 100 to 1,000 µmol m^−2^ s^−1^. A, Time to reach 95% of the steady-state *A* for five different banana genotypes. B, Percentage limitation of *A* after the increase in light intensity. Different letters indicate significant differences between genotypes (post hoc Tukey HSD test, *P* < 0.05; *n* = 7–8; A > B). The bold middle line in boxplots represents the median. The box is confined by the first and third quartile and the whiskers extend to 1.5 times the interquartile distance. Points falling outside the whiskers are considered outliers and plotted as dots.

The speed of the increase in *g_s_* following a step-change in light intensity from 100 to 1,000 µmol m^−2^ s^−1^ strongly determined CO_2_ uptake during this period. The speed of changes in *g_s_* in all genotypes accounted for ˃89% of *A* limitation (Supplemental Figure S3A). The time to reach 95% of steady-state *A* at 1,000 µmol m^−2^ s^−1^ (*A*_1,000_) was ˃30 min for almost all genotypes and differed significantly between Cachaco (51.9 min) and the genotypes Mbwazirume (30.3 min) and Banksii (29.5 min; Figure 3A;Supplemental Figure S4 and Supplemental Table S2). This timing of *A* limitation was significantly correlated with the time to reach 95%, 90%, and 50% of steady-state *g_s_* (*P* < 0.001, *R*^2^ = 0.42–0.48), while there was no significant relation with the time to reach 95% or 90% of the maximum carboxylation rate of Rubisco (*V*_cmax,_Supplemental Figures S1 and S3). The timing to reach 95% of steady-state *V*_cmax_ was ˂20 min in all genotypes, while the timing to reach 95% of steady-state *g_s_* was much longer and ranged between 41 and 69 min (Supplemental Figure S3 and Table S2). The durations of *A* limitation were also significantly correlated with the modeled time constant for *g_s_* increase (*K*_i_; *P* < 0.001, *R*^2^ = 0.67; Supplemental Figure S1). The percentage limitation of *A* was significantly higher in Cachaco (20.6%) compared to the genotypes Mbwazirume (10.2%), Leite (10.2%), and Banksii (8.5%; Figure 3B) and was significantly related to both *K*_i_ and the time to reach 90% and 50% of steady-state *g_s_*, confirming the impact of stomatal limitation on *A* (Supplemental Figure S1).

### iWUE response to step-changes in light intensity

The step increase in light intensity induced an initial increase in *A* that was relatively larger than the increase in *g_s_*. These responsiveness differences increased _i_WUE, reaching the maximum _i_WUE during the light period in all cases within 7.5 min (Supplemental Figure S5). After reaching a maximal value, _i_WUE decreased as both *g_s_* and *A* gradually increased (Supplemental Figure S5). _i_WUE only stabilized when both *A* and *g_s_* reached steady-state. The genotype Cachaco had a significantly higher mean _i_WUE during the high light period compared to Mbwazirume (Supplemental Figure S6). The mean _i_WUE during the high light period was significantly correlated with the time constant *K*_i_ and *Sl*_max,i_ with slower *g_s_* responses resulting in higher _i_WUE (*R*^2^ = 0.12 and 0.42, *P* < 0.05; Supplemental Figure S1). The reduction in light intensity from 1,000 to 100 µmol m^−2^ s^−1^ instantaneously lowered _i_WUE as *A* immediately declined because of light limitation (Supplemental Figure S5). The mean _i_WUE during this low light period was significantly higher in Kluai Tiparot, than in Leite (Supplemental Figure S6). The mean _i_WUE was significantly correlated to the stomatal closing variables *K*_d_ and *Sl*_max,d_ with faster *g_s_* responses resulting in higher _i_WUE (*R*^2^ = 0.36 and 0.26, *P* < 0.001; Supplemental Figure S1).

### Stomatal anatomy

**Figure 4:**
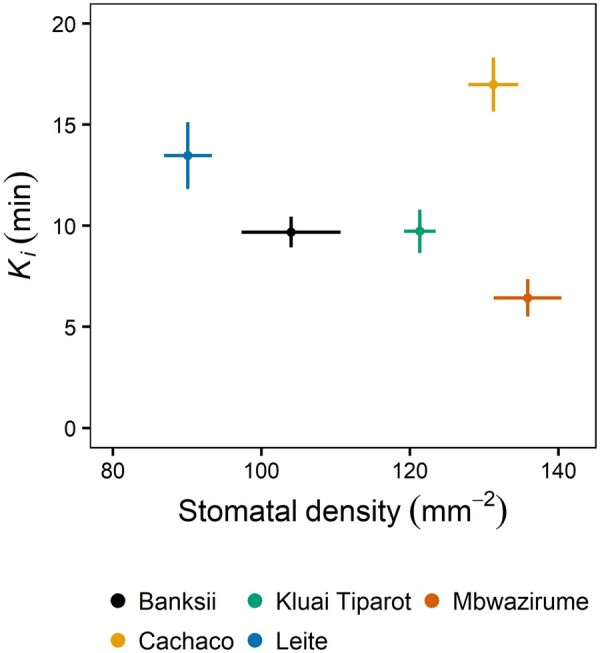
Relation between abaxial stomatal density and the time constant describing the speed of *g_s_* increase after the light intensity increase from 100 to 1,000 µmol m^−2^ s^−1^. There was no significant correlation (Pearson’s correlation test), caused by the outlying genotype Cachaco. Points and error bars represent mean ± se (*n* = 7–8).

Banana has elliptical-shaped guard cells surrounded by four to six subsidiary cells (Rudall et al., 2017). Abaxial stomatal density, stomatal length, guard cell size, and subsidiary cell size were quantified from the leaf part enclosed in the gas exchange cuvette and significant differences between genotypes were observed (Supplemental Figure S7). Stomatal density and stomatal length were not correlated with any of the modeled light-induced *g_s_* kinetics (Figure 4; Supplemental Figure S1). However, these correlations between anatomy and *g_s_* kinetics were significant if the genotype Cachaco with the lowest *g_s_* rapidity was not considered (Figure 4). In this case, stomatal density was significantly correlated with the time constant *K* as well as the maximum slope of *g_s_* response *Sl*_max_ during both stomatal opening and closing (*P* < 0.01; *R*^2^ = 0.25–0.46).

### Whole-plant transpiration response at dawn

**Figure 5:**
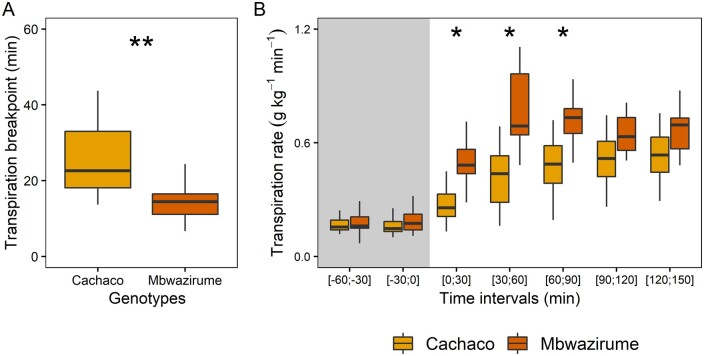
Gravimetric transpiration rate analysis of genotypes Cachaco and Mbwazirume at dawn after a step increase in light intensity from 0 to 120 µmol m^−2^ s^−1^. A, A breakpoint was identified in whole-plant transpiration after the step increase in light intensity. The timing of the breakpoint in transpiration after dawn differed significantly between the genotype Cachaco and Mbwazirume (*n* = 24, *P* < 0.01, linear mixed-effects model with plant-specific and date-specific random effect). B, Transpiration rate after dawn increased faster in Mbwazirume compared to Cachaco. Before dawn transpiration rates did not differ significantly. Similarly transpiration rates do not differ significantly after 90 min (24 datapoints per time range for both Cachaco and Mbwazirume, * for *P* < 0.05, ** for *P* < 0.01, linear mixed-effects model with plant-specific and date-specific random effect). Gray areas indicate the time before dawn. The bold middle line in boxplots represents the median The box is confined by the first and third quartile and the whiskers extend to 1.5 times the interquartile distance.

The significant differences in *g_s_* speed at leaf level observed between the two extreme genotypes Cachaco and Mbwazirume were confirmed at the whole-plant level under a step increase in light intensity from darkness (Figure 5) and under a gradually increasing light intensity (Supplemental Figure S8). After the onset of light in the morning, the transpiration rate increased significantly faster in Mbwazirume compared to Cachaco (Figure 5, A and B; Supplemental Figure S8A). After a step increase in light intensity, a significant increase in transpiration rate was observed after *c.* 15 min in Mbwazirume, while in Cachaco this was only after 25 min (Figure 5, A and B). Similar faster increases in transpiration rate of Mbwazirume were observed under a gradually increasing light intensity (Supplemental Figure S8A). The temporal response of whole-plant transpiration rate to a step increase in light intensity was also modeled following the sigmoidal model (Eq. 1) and the time constants *K*i differed significantly between genotypes (Supplemental Figure S9). Similar to the response at leaf level, Cachaco, had an average time constant *K*_i_ of 20 min, while Mbwazirume, had a *K*_i_ of 8.5 min (Supplemental Figure S9). The difference in transpiration responses was also reflected in the transpiration rate before and after dawn. The whole-plant transpiration rate did not differ significantly between both genotypes pre-dawn, but after the step change in light intensity, the transpiration rate was significantly higher in Mbwazirume for 90 min, whereafter both genotypes reached similar steady-state transpiration rates (Figure 5B). Likewise, the transpiration rate under gradually increasing light intensity did not differ pre-dawn, but was significantly higher in Mbwazirume after the onset of light (Supplemental Figure S8B).

### Impact of diurnal light fluctuations on *g_s_*, *A*, and _i_WUE

**Figure 6:**
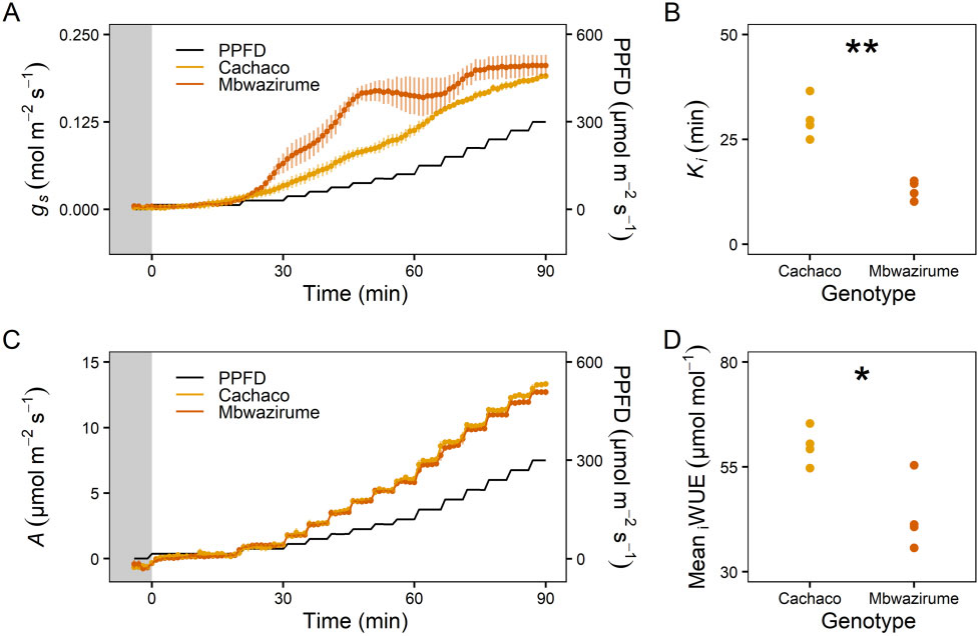
Morning response of *g_s_* and *A* of the genotypes Cachaco and Mbwazirume. A, Time course of the *g_s_* response to a gradual increase in light intensity at dawn (black line). Data are the mean ± se (*n* = 4). B, The time constant of *g_s_* increase (*K_i_*) during the first 90 min after dawn was significantly higher in Cachaco. C, The difference in *g_s_* rapidity at dawn did not result in different *A* between both genotypes. Data represent the mean ± se (*n* = 4). D, The mean _i_WUE during the first 90 min after dawn was significantly higher in Cachaco compared to Mbwazirume. The gray area indicates the time before dawn. (Student’s *t* test, *P < 0.05, ** *P* < 0.01).

**Figure 7:**
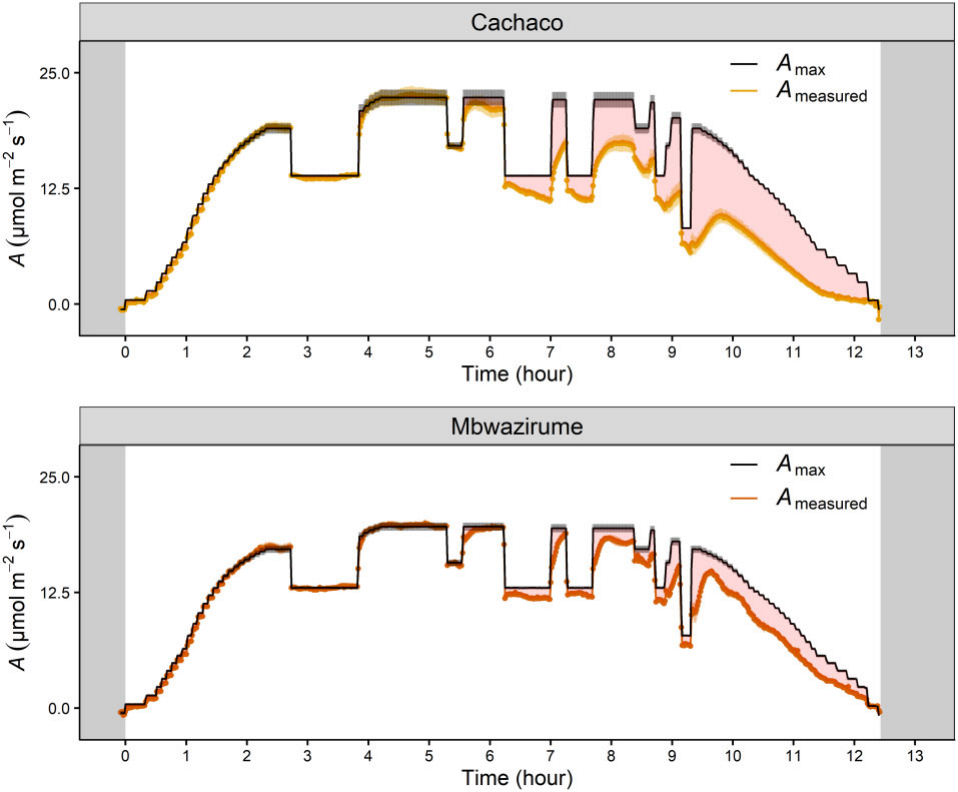
Mean diurnal time course of measured *A* (*A*_measured_ and maximal *A* (*A*_max,_ black line) under fluctuating light conditions for Mbwazirume and Cachaco. The *A*_max_ at each light intensity was determined by a modeled light response curve. The nonrectangular hyperbola-based model of Prioul and Chartier (1997) was optimized as described by Lobo et al. (2013). Grey areas indicate times of darkness, red areas indicate the difference between maximal *A* and measured *A*. Data are the mean ± se (*n* = 4).

To evaluate the impact of *g_s_* kinetics on diurnal *A* and _i_WUE, plants were subjected to fluctuating light intensities and phenotyped over an entire diurnal period. Similar to the transpiration rate measured at the whole-plant level, the morning increase in *g_s_* at leaf-level under gradually increasing light intensity was faster in Mbwazirume compared to Cachaco (Figure 6A). The time constant for the *g_s_* increase (*K*_i_) was significantly higher in Cachaco (*P* < 0.005; Figure 6B). However, the faster increase of *g_s_* in Mbwazirume, did not result in increased *A* (Figure 6C)*.* Maximum potential *A* values at specific light intensities were determined from light response curves and compared to those measured under the diurnal conditions. Under the gradual increasing light intensities experienced in the morning, maximum *A* values were achieved, indicating there was no *g_s_* limitation under these light-limiting conditions (Figure 7). A similar *A* with lower *g_s_* during the morning, led to a significantly higher mean _i_WUE in Cachaco (*P* < 0.05, Figure 6D).

**Figure 8:**
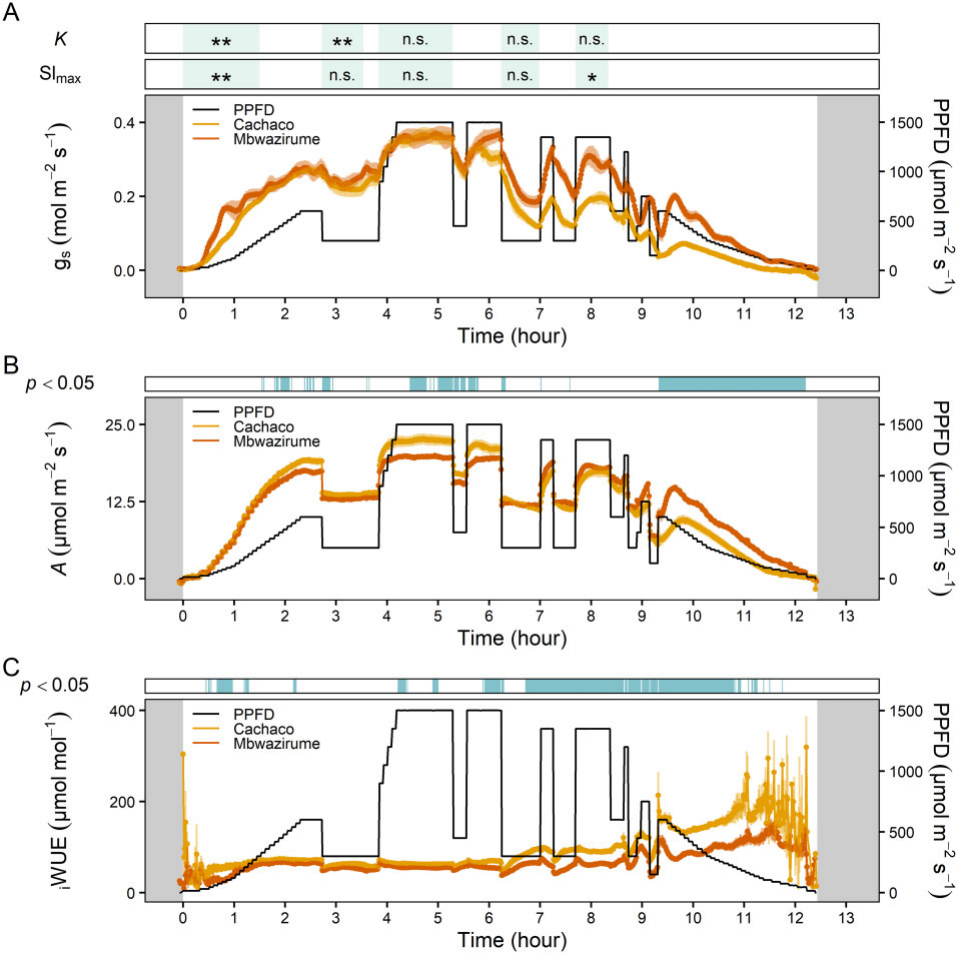
Diurnal time course of gas exchange parameters of the genotypes Mbwazirume and Cachaco under fluctuating light conditions. A, *g_s_*, (B) *A*, and (C) _i_WUE. The light intensity fluctuated throughout the day (black line). The significance of the time constant of *g_s_* increase or decrease (*K*) and the maximal slope of *g_s_* increase or decrease (*Sl*_max_) is shown (Student’s *t* test, *P < 0.05 and **P < 0.01 for faster *g_s_* rapidity in Mbwazirume compared to Cachaco). Throughout the day, *g_s_* kinetics were faster for the genotype Mbwazirume compared to Cachaco, but differences were dependent on the target light intensity, the magnitude of change, the *g_s_* prior to the intensity change, and the time of the day. Gray areas indicate times of darkness. Green areas indicate the analyzed time frame of the *g_s_* rapidity response. Blue areas indicate time points with significant differences in *A* or _i_WUE between both genotypes (Student’s *t* test, *P* < 0.05). Data are the mean ± se (*n* = 4).

**Figure 9:**
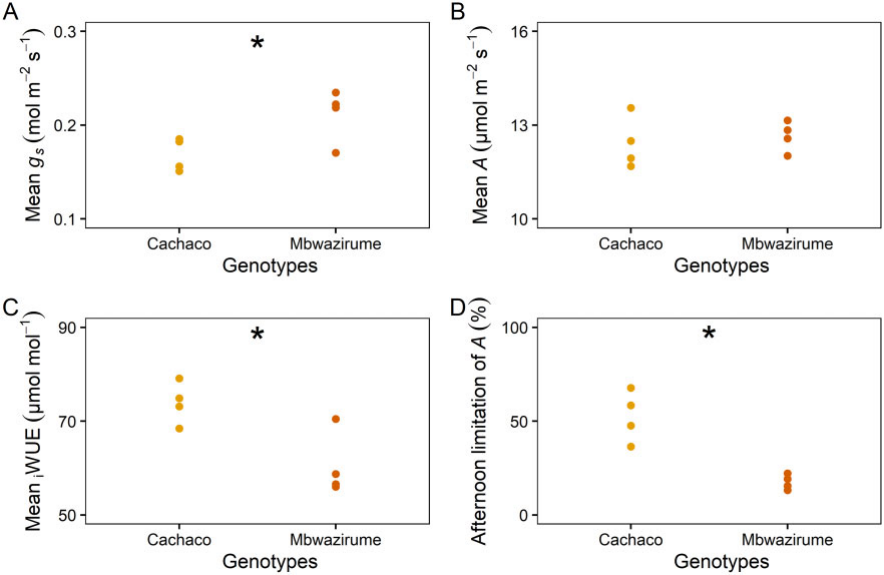
Average diurnal gas exchange parameters of the genotypes Mbwazirume and Cachaco under fluctuating light conditions illustrated in Figure 8. A, *g_s_*, (B) *A*, and (C) iWUE. D, The percentage limitation of *A* during the afternoon (>6 h after light onset). (Student’s *t* test, * *P* < 0.05, *n* = 4).

Throughout the day, *g_s_* kinetics were in most cases significantly faster for the genotype Mbwazirume compared to Cachaco (Figure 8A), again confirming the previously observed kinetics (Figures 2 and 5). However, under fluctuating light conditions *g_s_* kinetics were dependent on the magnitude of light intensity change, *g_s_* values prior to the light intensity change, and the time of the day (Figure 8A). During the afternoon, there was a setback in kinetics: the absolute *g_s_* and the *g_s_* responses to light were damped (Figures 7 and 8). Simultaneously, *A* decreased greatly in the afternoon, which could be mainly attributed to a reduction in *g_s_*. The limitation of *A* in the afternoon was 3 times higher in Cachaco (52.6%) compared to Mbwazirume (17.5%; Figures 7 and 9D). The reduction of *g_s_* in the afternoon resulted in a significantly lower average diurnal *g_s_* (Figure 9A) which translated into a greater diurnal _i_WUE in Cachaco compared to Mbwazirume (Figures 8C and 9C).

## Discussion

### Stomatal behavior greatly limits *A* in banana

Step changes in light intensity have been shown to induce an uncoupling of *A* and *g_s_* in many species (Barradas and Jones, 1996; Lawson and Blatt, 2014; McAusland et al., 2016; Faralli et al., 2019a). However, all banana genotypes maintain a tight coupling between *A* and *g_s_* following a step increase in light intensity (Figure 1). This indicates a strong stomatal control of *A*, which is demonstrated by diffusional limitations accounting for ˃89% of *A* limitation (Supplemental Figure S3A). This high stomatal limitation of *A* is explained by the slow *g_s_* response (Figures 1 and 2) relative to the faster biochemical activation. The time required for biochemical activation was much lower and not correlated with the time for steady-state *A* and *g_s_* (Supplemental Figure S3). Similar to Deans et al. (2019a) and De Souza et al. (2020), the speed of changes in *g_s_* was the predominant limitation of *A*. This behavior shows that banana strongly controls stomatal aperture, resulting in water conservation at the expense of potential carbon gain, which supports the early work of Aubert and Catsky (1970). This prioritizing of water conservation in banana can be explained by its intrinsic need to maintain a high leaf water potential (Turner and Thomas, 1998).

### Diversity in light-induced stomatal responses

Stomatal responses to changes in light intensity have been shown to vary at an inter- and intra-specific level (Vico et al., 2011; Drake et al., 2012; McAusland et al., 2016; Qu et al., 2016; De Souza et al., 2020; Durand et al., 2020). A higher steady-state *g_s_* has been linked with faster light-induced *g_s_* responses (Drake et al., 2012; Kaiser et al., 2016; McAusland et al., 2016; Wachendorf and Küppers, 2017; Sakoda et al., 2020). Although the differences observed in steady-state *g_s_* values between banana genotypes were not significant, their *g_s_* kinetics differed strongly (Figure 2). These results suggest that other factors such as stomatal anatomy, hydraulic conductance and membrane transporters are involved in determining the rapidity of changes in *g_s_*.

The banana B genome is often related to drought tolerance because of its center of origin and its natural occurrence in drier habitats under full sunlight (Perrier et al., 2011; Janssens et al., 2016; Eyland et al., 2021). Within the investigated banana genotypes, we observed significant differences in the speed of increase and decrease in *g_s_* (Figure 2, B and C). However, differences across genotypes were not explained by their genomic constitution (see “Materials and Methods” section), which is in agreement with the wide diversity of transpiration phenotypes observed irrespective of genomic constitution (van Wesemael et al., 2019).

Consistent with previous works in other species (Vico et al., 2011; McAusland et al., 2016; Faralli et al., 2019a), the speed of *g_s_* increase and decrease was significantly correlated (Figure 2D). Decreases in *g_s_* were faster than opening in all banana genotypes (Figure 2D), which is not the case for all crops (McAusland et al., 2016; Qu et al., 2016). The faster *g_s_* closure again indicates that banana prioritizes water conservation over maximization of carbon uptake.

The two most extreme genotypes Cachaco and Mbwazirume, with the slowest and fastest *g_s_* responses, respectively, also showed at the whole-plant level differences in the light-induced speed of transpiration rate increase (Figure 5; Supplemental Figures S8 and S9). This finding suggests that despite possible differences in *g_s_* control of water loss at different locations of the leaf (Matthews et al., 2017) and across leaves of different ages (Urban et al., 2008) genotype-specific responses are still maintained. Leaf-level measurements of light-induced *g_s_* kinetics are thus in line with whole-plant responses. To our knowledge, this is the first report confirming stomatal kinetics at the whole-plant level. The genotype-specific difference in whole-plant transpiration responses at dawn was validated at the leaf level with *g_s_* increasing faster in Mbwazirume under gradually increasing light intensity (Figure 6A). This faster *g_s_* increase in Mbwazirume did not result in higher *A*, indicating that at dawn, under gradually increasing low light intensities, *g_s_* was not limiting *A* and was higher than necessary for maximal *A* (Figures 6 and 7). These results demonstrate that the impact of *g_s_* kinetics on *A* and _i_WUE depends on the time of the day and the light conditions. The uncoupling of *g_s_* and *A* under increasing light conditions at dawn was not beneficial for carbon uptake. Gosa et al. (2019) called this period after dawn in tomato the golden hour because in dry climates it is the time of the day with the highest *g_s_*. Later in the day, VPDs become too high, restricting *g_s_* (Gosa et al., 2019). Breeding for an even higher *g_s_* during this golden hour was suggested to improve plant productivity. However, care must be taken to breed for an improved morning CO_2_ uptake, rather than for a high *g_s_* with associated uncoupling of *A* and *g_s_*. Although the absolute water loss resulting from excessive morning *g_s_* might be relatively low because of low evaporative demands at dawn (Chaves et al., 2016), it may lead to a crucial decrease in overall plant water status.

Despite the confirmed genotypic differences in stomatal kinetics, the impact of *g_s_* kinetics on *A* and _i_WUE before noon hardly differed between the genotypes Cachaco and Mbwazirume under field-mimicking light conditions (Figures 7 and 8). This could be explained by lower amplitudes of light switches compared to a single step change in light intensity and/or *g_s_* values not being at steady-state prior to changing light intensity. The genotype-specific speed of the *g_s_* response observed under a single step change in light intensity did not explain the diurnal _i_WUE, indicating that *g_s_* kinetics only partially affect diurnal WUE and carbon gain (Figure 9, B and C). The absolute *g_s_* and the *g_s_* responses to light decreased strongly in the afternoon, and this effect was more pronounced in the genotype Cachaco (Figures 7 and 8A). The 3 times higher afternoon limitation of *A* in the genotype Cachaco compared to Mbwazirume, resulted in a significantly higher diurnal _i_WUE (Figure 9, C and D). The genotype Cachaco with the slowest *g_s_* kinetics thus achieved the highest _i_WUE, showing that not only *g_s_* speed but also the *g_s_* diurnal pattern determines the overall WUE and carbon gain. Although the mechanism behind the afternoon *g_s_* reduction remains largely unknown, it is commonly hypothesized to be related to circadian regulation of ABA sensitivity and associated endogenous signals regulating the clock, such as feedback loops from photosynthate accumulation (Mencuccini et al., 2000; Haydon et al., 2013; Delorge et al., 2014; Resco de Dios and Gessler, 2018). We show that under fluctuating light conditions this intrinsic diurnal pattern of absolute *g_s_* decrease and *g_s_* light responsivity reduction is decisive for diurnal _i_WUE (Figure 9C).

### Impact of stomatal anatomy on responses

Stomatal density, as well as the size, have been reported to affect *g_s_* kinetics (Hetherington and Woodward, 2003; Drake et al., 2012; Raven, 2014; Sakoda et al., 2020). However, McAusland et al. (2016) and Faralli et al. (2019a) reported no or only a weak inter- and intra-specific correlation between stomatal anatomy and light-induced *g_s_* kinetics. We confirmed that stomatal density and size were not correlated with the *g_s_* kinetics (Figure 4; Supplemental Figure S1). Remarkably, the genotype with the slowest increase in *g_s_*, Cachaco had the second highest density and the smallest stomata. Without this genotype a significant correlation between density and the speed of *g_s_* increase and decrease was observed (Figure 4). This exception suggests that the surface-to-volumes ratios are not always directly related to stomatal speed as this assumes uniform ion transport activity per surface area (Lawson and Blatt, 2014).

## Conclusion

Our findings show that there is diversity in *g_s_* rapidity to light within closely related banana genotypes and that slow stomatal responses and not biochemical activation greatly limit *A*. The priority of banana for water saving is shown by strong stomatal control of *A* and faster decrease in *g_s_* than increase. The observed diversity in *g_s_* rapidity was not related to stomatal anatomy and therefore suggests that variation is rather driven by functional components. We show here for the first time that the *g_s_* rapidity observed at the leaf level can also be found at the whole-plant level. However, under fluctuating light conditions, *g_s_* rapidity is only one of the many physiological factors determining overall plant WUE and carbon gain.

## Materials and methods

### Experiment 1: Leaf gas exchange response to a step-change in light intensity

#### Plant material and growth conditions

Banana plants (*Musa* spp.) were obtained through the International Musa Transit Center (ITC, Bioversity International), hosted at KU Leuven, Belgium. Plants of five genotypes from different subgroups were selected: Banksii (subgroup Banksii, AA genome, ITC0623), Cachaco (Bluggoe, ABB genome, ITC0643), Kluai Tiparot (Kluai Tiparot, ABB genome, ITC0652), Leite (Rio, AAA genome, ITC0277), and Mbwazirume (Mutika-Lujugira, AAA genome, ITC1356). Plants were grown in 800 mL containers filled with peat-based compost (Levingtons F2S, UK) under 350 µmol m^−2^ s^−1^ photosynthetic photon flux density (PPFD) in a 12-h: 12-h light: dark cycle with temperature and relative humidity at 26 ± 1°C and 70 ± 10%, respectively. Plants were well-watered and starting from Week 3 a Hoagland nutrient solution was added. Measurements were performed when plants were fully acclimated and 7 weeks old.

#### Leaf gas exchange measurements

*A* and *g_s_* to water were measured every 30 s on the middle of the second youngest fully developed leaf using an LI-6400XT infrared gas analysis and dew-point generator model LI-610 (LI-COR, Lincoln, NE, USA). Light was applied by an integrated LED light source. The leaf cuvette maintained a CO_2_ concentration of 400 µmol mol^−1^, a leaf temperature of 25°C, and a VPD of 1 kPa. All measurements were performed before 14:00 h to avoid circadian influences.

#### Stomatal response to a step change in light intensity

The light intensity was kept at 100 µmol m^−2^ s^−1^ until *A* and *g_s_* were stable for 10 min. Once steady-state was reached, light intensity was increased to 1,000 µmol m^−2^ s^−1^ for 90 min. Then, light intensity was lowered back to 100 µmol m^−2^ s^−1^ for 30 min.

The increase in *g_s_* after the increase in light intensity and the decrease in *g_s_* after the decrease in light intensity followed a sigmoidal pattern and was modeled using the nonlinear sigmoidal model described in Vialet-Chabrand et al. (2017):

(Eq. 1) $$g_{s}=\;(g_{s,1000}-g_{s,100})\;e^{-e(\frac{\lambda -t}{K}+1)}+\;g_{s,1000}$$

With *g_s_* the *g_s_* at time t, *K* the time constant for rapidity of *g_s_* response (min), λ the lag time of the sigmoidal curve (min), *g_s_*_,100_ and *g_s_*_,1,000_ (mol m^−2^ s^−1^) the steady-state *g_s_* at 100 and 1,000 µmol m^−2^ s^−1^, respectively. Parameter values were estimated for each individual plant using nonlinear model optimization in R version 3.4.3. *K*_i_ indicates the *g_s_* increase time constant, *K*_d_ the *g_s_* decrease time constant. The maximum slope of *g_s_* during opening and closing was calculated and defined as *Sl*_max_. _i_WUE was calculated as _i_WUE = *A*/*g_s_*. Outlying values (0.5% quantile; _i_WUE < 0 or >400 µmol mol^−1^) caused by low *g_s_* were discarded for plotting.

#### Stomatal and biochemical limitation analysis

*A* was considered to be limited until 95% of steady-state *A* at 1,000 µmol m^−2^ s^−1^ was reached (McAusland et al., 2016). The percentage of limitation of *A* was calculated by comparing the measured *A* with the maximal steady-state *A* under 1,000 µmol m^−2^ s^−1^ according to McAusland et al. (2016):

(Eq. 2) $$\mathrm{Limitation}\;\mathrm{of}\;A\;(\%)\;=\int_{0}^{t}\sum (A_{\max}-A_{\mathrm{measured}})\int_{0}^{90}\sum A\mathrm{measured}$$

With *A*_max_ the value reached at 95% of steady-state *A* under 1,000 µmol m^−2^ s^−1^, *A*_measured_ the measured *A* and *t* the time where 95% of steady-state *A* is reached.

The delay in obtaining maximum potential *A* under 1,000 µmol m^−2^ s^−1^ is determined by the stomatal opening speed as well as the rate of biochemical activation. The activation rate of Rubisco is the main biochemical limiting component during step changes in light exceeding several minutes (Mott and Woodrow, 2000; Way and Pearcy, 2012). To quantify the relative contributions of biochemical and stomatal limitations a differential method was applied (Jones, 1985; Wilson et al., 2000; Grassi and Magnani, 2005; Deans et al., 2019b). As explained by Deans et al. (2019b), the forgone *A* because of biochemical and stomatal limitation was calculated as:

(Eq. 3) $$dA_{\mathrm{biochem}}=\partial A\partial V_{\mathrm{cmax}}{\mathrm{dV}}_{\mathrm{cmax}}$$

and

(Eq. 4) $$dA_{\mathrm{stom}}=\partial A\partial g_{\mathrm{sc}}dg_{\mathrm{sc}}$$

where *V*_cmax_ is the maximum velocity of Rubisco for carboxylation and *g_s_*_c_ the *g_s_* to CO_2_. *V*_cmax_ at every time point was calculated by solving the Rubisco-limited *A* as described by Farquhar et al. (1980) for *V*_cmax_:

(Eq. 5) $$V_{\mathrm{cmax}}=(A+R_{d})(C_{i}+K_{m})(C_{i}-\Gamma^{*})$$

where *C*_i_ is the CO_2_ concentration in the intercellular airspaces of the leaf. *R*_d_ represents the mitochondrial respiration for which average dark respiration rates were used. Γ* is the photorespiratory compensation point and *K*_m_ is the effective the Rubisco Michaelis–Menten constant for CO_2_ under 21% O_2_. Values for Γ* and *K*_m_ were taken as the average for C3 species at 25°C as described by Hermida-Carrera et al. (2016), 41.2 and 529.4 µmol mol^−1^, respectively. Mesophyll conductance to CO_2_ was assumed to be infinite. *g_s_*_c_ at every time point was calculated as:

(Eq. 6) $$g_{\mathrm{sc}}=\frac{g_{s}}{1.6}$$

The relative stomatal limitation (σ_stom_) was then calculated as:

(Eq. 7) $$\sigma_{\mathrm{stom}}=\int_{0}^{t}\text{d}A_{\mathrm{stom}}\mathrm{dt}\int_{0}^{t}\text{d}A_{\mathrm{biochem}}\mathrm{dt}\int_{0}^{t}\text{d}A_{\mathrm{stom}}dt$$

where *t* represents the time where 95% of steady-state *A* under 1,000 µmol m^−2^ s^−1^ was reached. Timings representing the *g_s_* and *A* increase were calculated at 95%, 90%, and 50% of steady-state values under 1,000 µmol m^−2^ s^−1^. Timings for *V*_cmax_ were calculated at 95% and 90% of steady-state values.

#### Stomatal anatomy measurements

Stomatal impressions of the abaxial surface of the leaf were made when stomata were completely closed using impression material. Impression was made by applying dental polymer according to the protocol of Weyers and Johansen (1985), followed by covering the polymer with nail varnish and placement on a microscope slide. Impressions were only taken from the abaxial side, because stomatal densities are generally 75% higher compared to the adaxial side in banana, therefore majorly determining gas exchange as shown by Brun (1961). Stomatal anatomy was quantified using an EVOS digital inverted microscope. Stomatal density was determined in three microscopic fields of views of 1.12 mm^2^ captured with a 10× objective lens (54–117 stomata per field of view). Guard cell length (µm), guard cell size (mm^2^), and lateral subsidiary cell size (mm^2^) were determined in three microscopic field of views of 0.07 mm^2^ captured with a 40× magnification, respectively (four to seven stomata per field of view). Measurements were performed in ImageJ software (http://rsb.info.nih.gov/ij).

### Experiment 2: Whole-plant transpiration response at dawn

#### Plant material and growth conditions greenhouse experiment

For the genotypes Cachaco and Mbwazirume, 12 plants were grown for 7 weeks in a greenhouse prior to the experiment. Plants were grown in 10 L containers filled with peat-based compost. At the start of the experiments, the six most homogenous plants per genotype were selected based on leaf area. Weight of each plant was followed by a multi-lysimeter setup of high precision balances, registering the weight every 60 s (1 g accuracy, Phenospex, Heerlen, Netherlands). The soil was covered by plastic to avoid evaporation and ensure only waterloss through transpiration. The transpiration rate was calculated by differentiating the raw weight data over time. The soil water content was determined by subtracting the plastic pot weight, the dry soil weight, and the plant weight from the total weight measurement. Dry soil weight was calculated as a function of the soil volume (bulk density = 0.2267 g cm^–^³). Leaf area was calculated by weekly top view imaging and model over time by a power-law function (Paine et al., 2012):

(Eq. 8) $$\mathrm{leaf}\;\text{area}\;=\text{k}\;+\;a*\mathrm{day}s^{b}$$

The daily plant weight was estimated from the projected leaf area using genotype-specific correlations (*n* > 50; *R*^2^ ≥ 0.94). Plants were watered with a nutrient solution during the night and kept at well-watered conditions. Radiation was collected every 5 min via a sensor (Skye instruments, Llandrindod Wells, UK) inside the greenhouse. Supplemental lighting of 14 W m^−2^ at plant level was provided when solar radiation was ˂250 W m^−2^ during the daytime. Temperature and relative humidity data were collected using six data loggers (Trotec, Heinsberg, Germany ) registering data every 5 min. The onset of light was defined as the moment when intensity increased ˃2 W m^−2^.

#### Plant material and growth conditions controlled environment experiment

For the genotypes Cachaco and Mbwazirume, three plants were grown in a growth chamber with relative humidity of 70% and temperature of 24°C. Plants were grown hydroponically in containers with 350 mL medium (see van Wesemael et al. (2019) for specific nutrient composition) and placed under adjustable LED panels (LuminiGrow 600R1; Lumini technology Co. Ltd., Zhejiang, China) providing 120 µmol m^−2^ s^−1^ in a 12/12-h light/dark cycle. Plants were 5 weeks old at the start of the experiment and weighted prior to the experiment to normalize for plant mass. Biomass was again measured after 8 d, at the end of the experiment. Water loss of each plant was followed by a multi-lysimeter setup of high precision balances (0.01 g accuracy; Kern, Balingen, Germany). Balances were connected to a computer registering the weight every 10 s.

### Experiment 3: Impact of diurnal light fluctuations on *g_s_*, *A*, and _i_WUE

#### Plant material and growth conditions

Four plants of the genotypes Cachaco and Mbwazirume were grown in a greenhouse. Plants were grown in 4 L containers filled with peat-based compost and maintained under well-watered conditions. After 8 weeks plants were moved to a growth chamber with relative humidity 70 ± 15% and temperature 28 ± 2°C.

#### Leaf gas exchange measurements

*A* and *g_s_* were measured every minute on the middle of the second youngest fully developed leaf using an LI-6800 infrared gas analyzer (LI-COR, Lincoln, NE, USA). The leaf cuvette maintained a CO_2_ concentration of 400 µmol mol^−1^, a leaf temperature of 28°C and a VPD of 1 kPa. The light intensity was programmed to fluctuate throughout the day. Plants were placed under adjustable LED panels (LuminiGrow 600R1; Lumini technology Co. Ltd.,Zhejiang, China) that mimicked light fluctuations inside the LI-6800 leaf cuvette. The *g_s_* response was described using the nonlinear sigmoidal model of Vialet-Chabrand et al. (2013) where light or dark steps were sufficiently long for model optimization (Eq. 1). A light response curve with *A* in function of PPFD was modeled for each individual based on *A* values recorded during the first 6 h of the day that was not limited by *g_s_*. The nonrectangular hyperbola-based model of Prioul and Chartier (1977) was optimized as described C Lobo et al. (2013):

(Eq. 9) $$A=\;\mathrm{PPFD}*\Phi_{0}+A_{\max}-\sqrt{\Phi_{0}*\mathrm{PPFD}+A_{\max}2-4\theta *\Phi_{0}*\mathrm{PPFD}*A_{\max}}2\theta -R_{n}$$

With *A* the photosynthetic rate (µmol m^−2^ s^−1^), PPFD (µmol m^−2^ s^−1^), Φ_0_ the quantum yield at PPFD of 0 µmol m^−2^ s^−1^ (µmol µmol^−1^), *A*_max_ the absolute maximum photosynthetic rate (µmol m^−2^ s^−1^), θ the dimensionless convexity factor and *R*_n_ the dark respiration (µmol m^−2^ s^−1^).

The percentage of limitation of *A* by *g_s_* during the afternoon (>6 h after light onset) was calculated by estimating the maximal potential *A* without *g_s_* limitation and comparing it with the measured *A* (Eq. 2).

### Statistical analysis and data processing

All data processing and statistical analysis were carried out in R version 3.4.3. Genotypic differences were tested by applying one-way analysis of variance with a post hoc Tukey HSD test. Segmented regression was performed on the whole-plant transpiration between −90 and 90 min relative to the onset of light. Data with no significant segmented regression (*P*-value Davies Test <0.05, segmented R package, 7.5% of the data) and negative slopes (2.5% of the data) were removed. Transpiration rate was calculated as the mean water loss every 30 min. To use the sigmoidal model (1) on whole-plant transpiration data, 1 min weight measurements were smoothed according to the Savitzky and Golay (1964) method with a filtering window of 21 and a fourth-order polynomial. Each day of whole-plant transpiration responses was regarded as a new replicate by incorporating a plant-specific factor and a date-specific factor as a random effect in a linear mixed model.

## Supplemental data

The following materials are available in the online version of this article.

**Supplemental Figure S1.** Correlation matrix of gas exchange and stomatal anatomy variables.

**Supplemental Figure S2.** Maximum slope of *g_s_* response (Sl_max_) to an increase in light intensity from 100 µmol m^−2^ s^−1^ to 1,000 µmol m^−2^ s^−1^ and to a decrease in light intensity from 1,000 µmol µmol m^−2^ s^−1^ to 100 µmol m^−2^ s^−1^

**Supplemental Figure S3.** Stomatal limitation of *A* and timings until steady-state values of *gs*, *A*, and maximum velocity of Rubisco for carboxylation (*V*_cmax_) were reached after an increase in light intensity from 100 µmol m^−2^ s^−1^ to 1,000 µmol m^−2^ s^−1^.

**Supplemental Figure S4.** Increase in *A* after increasing the light intensity from 100 µmol m^−2^ s^−1^ to 1,000 µmol m^−2^ s^−1^. A was considered limited until 95% of steady-state *A* was reached.

**Supplemental Figure S5.** Response of iWUE and the intracellular CO2 to a step increase and decrease in light intensity from 100 to 1,000 µmol m^−2^ s^−1^ and back.

**Supplemental Figure S6.** Mean iWUE after the increase in light intensity from 100 to 1,000 µmol m^−2^ s^−1^ and the decrease to 100 µmol m^−2^ s^−1^ afterward.

**Supplemental Figure S7.** Stomatal density, stomatal length, guard cell size, subsidiary cell size, and proportion of subsidiary cells of the five banana genotypes.

**Supplemental Figure S8.** Gravimetric transpiration rate analysis of genotypes Cachaco and Mbwazirume under gradual increasing light intensity.

**Supplemental Figure S9.** Modeled time constant (Ki) for the whole-plant transpiration rate increase of genotypes Cachaco and Mbwazirume after a step increase in light intensity from 0 to 120 µmol m^−2^ s^−1^.

**Supplemental Table S1.** Modeled steady-state and light-induced variables of the *gs* response to a step increase and decrease in light intensity from 100 to 1,000 µmol m^−2^ s^−1^ for five different banana genotypes.

**Supplemental Table S2.** Time to reach 95%, 90%, and 50% of steady-state *A*, *gs*, and *V*_cmax_ after a step increase in light intensity from 100 to 1,000 µmol m^−2^ s^−1^ for five different banana genotypes.

## Supplementary Material

kiab114_Supplementary_Data

## Notes

S.C. and T.L. supervised the experiments. D.E. performed the leaf gas exchange experiments. J.v.W. performed the whole-plant transpiration experiment. D.E., J.v.W., and S.C. analyzed the data. D.E., T.L., and S.C. wrote the manuscript. All authors reviewed and approved the final manuscript.

The author responsible for distribution of materials integral to the findings presented in this article in accordance with the policy described in the Instructions for Authors (https://academic.oup.com/plphys/pages/general-instructions) is: Sebastien Carpentier (s.carpentier@cgiar.org).
