# Supplementary material for: The impact of slow stomatal kinetics on photosynthesis and water use efficiency under fluctuating light
Source: Plant Physiol. 2021 Mar 8;186(2):998–1012. doi: 10.1093/plphys/kiab114 (PMC8195518; doi:10.1093/plphys/kiab114)
Supplement: kiab114_Supplementary_Data [file kiab114_supplementary_data.pdf]

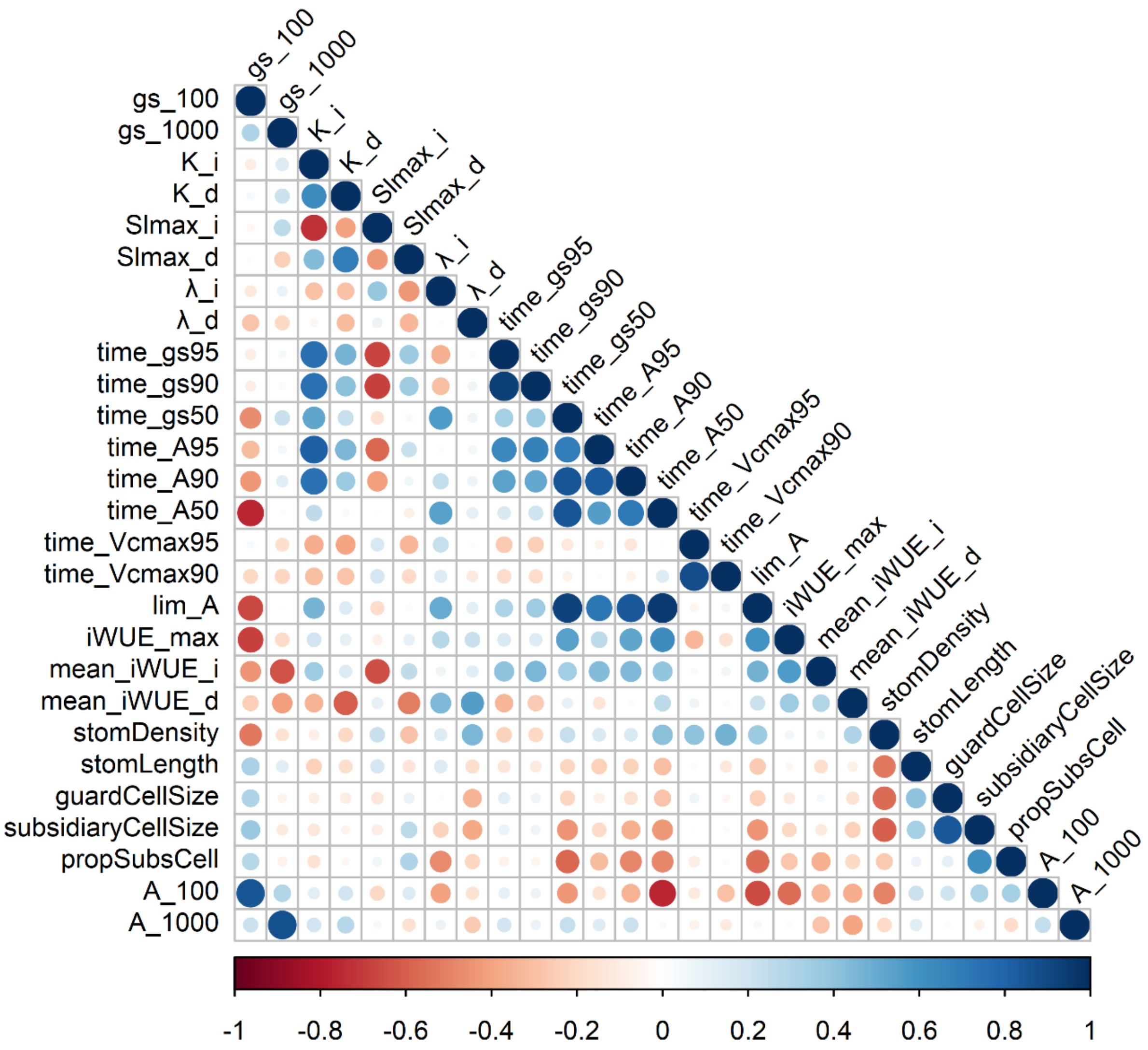

Supplemental Fig. S1 Correlation matrix of gas exchange and stomatal anatomy variables. Colors indicate correlation coefficient with blue strong positive correlations and red strong negative correlations (Pearson's correlation). gs_100, steady-state stomatal conductance (g_s) under 100 µmol m^{-2} s^{-1}; gs_1000, steady-state g_s under 1000 µmol m^{-2} s^{-1}; K_i, stomatal opening constant; K_d, stomatal closing constant; Slmax_i, maximum slope of stomatal conductance during opening; Slmax_d, maximum slope of stomatal conductance during closing; λ_i the initial time lag during stomatal opening; λ_d the initial time lag during stomatal opening; time_gs95, the time to 95% of steady-state g_s under 1000 µmol m^{-2} s^{-1}; time_gs90, the time to 90% of steady-state g_s; time_gs50, the time to 50% of steady-state g_s; time_A95, the time to 95% of steady-state photosynthesis (*A*) under 1000 µmol m^{-2} s^{-1}; time_A90, the time to 90% of steady-state *A* ; time_A50, the time to 50%

of steady-state *A*; time_Vcmax95, the time to 95% of maximum velocity of Rubisco for carboxylation (V_{cmax}) under 1000 µmol m^{-2} s^{-1}; time_Vcmax90, the time to 90% of V_{cmax} ; lim_A, limitation of *A* under 1000 µmol m^{-2} s^{-1} ; iWUE_max, maximum iWUE reached during the stomatal opening response; cumulative_iWUE_i, cumulative iWUE during stomatal opening; cumulative_iWUE_d, cumulative iWUE during stomatal closing; stomDensity; abaxial stomatal density; stomLength, length of stomatal guard cells; guardCellSize, size of the guard cells; subsidiaryCellSize, size of the lateral subsidiary cells; propSubsCell, proportion of subsidiary cells in the stomatal complex; A100, steady-state photosynthesis at 100 µmol m^{-2} s^{-1}; A1000, steady-state photosynthesis at 1000 µmol m^{-2} s^{-1}.

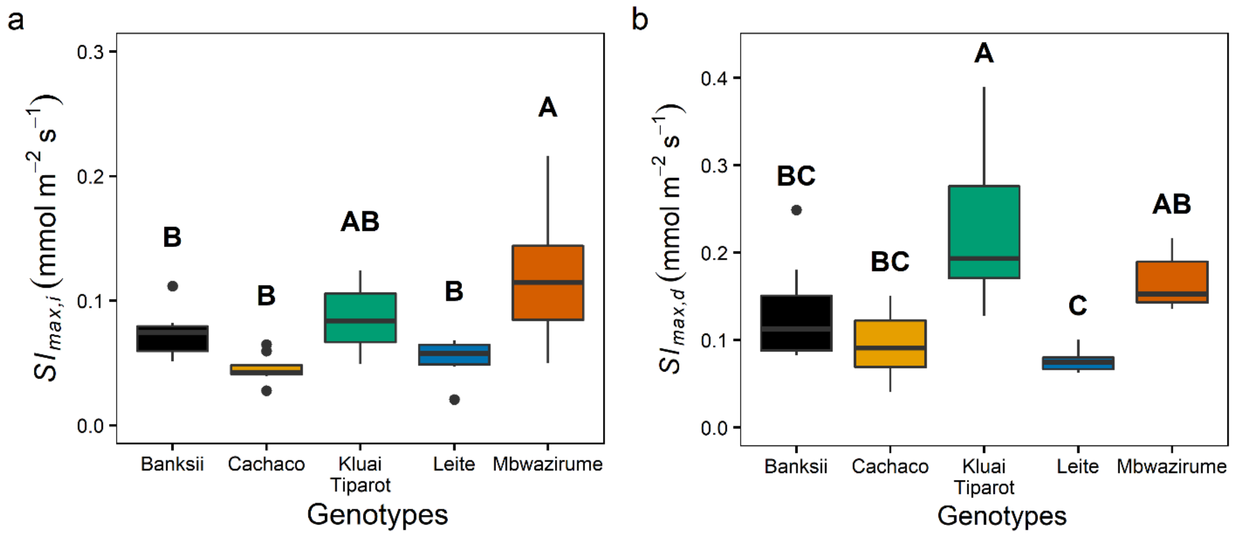

Supplemental Fig. S2 Maximum slope of stomatal conductance response (Sl_{max}) (a) to an increase in light intensity from 100 µmol m^{-2} s^{-1} to 1000 µmol m^{-2} s^{-1} and (b) to a decrease in light intensity from 1000 µmol m^{-2} s^{-1} to 100 µmol m^{-2} s^{-1}. Different letters indicate significant differences between genotypes (post hoc Tukey HSD test, $P < 0.05$, A>B>C, n = 7-8). The bold middle line in boxplots represents the median. The box is confined by the first and third quartile and the whiskers extend to 1.5 times the interquartile distance. Points falling outside the whiskers are considered outliers and plotted as dots.

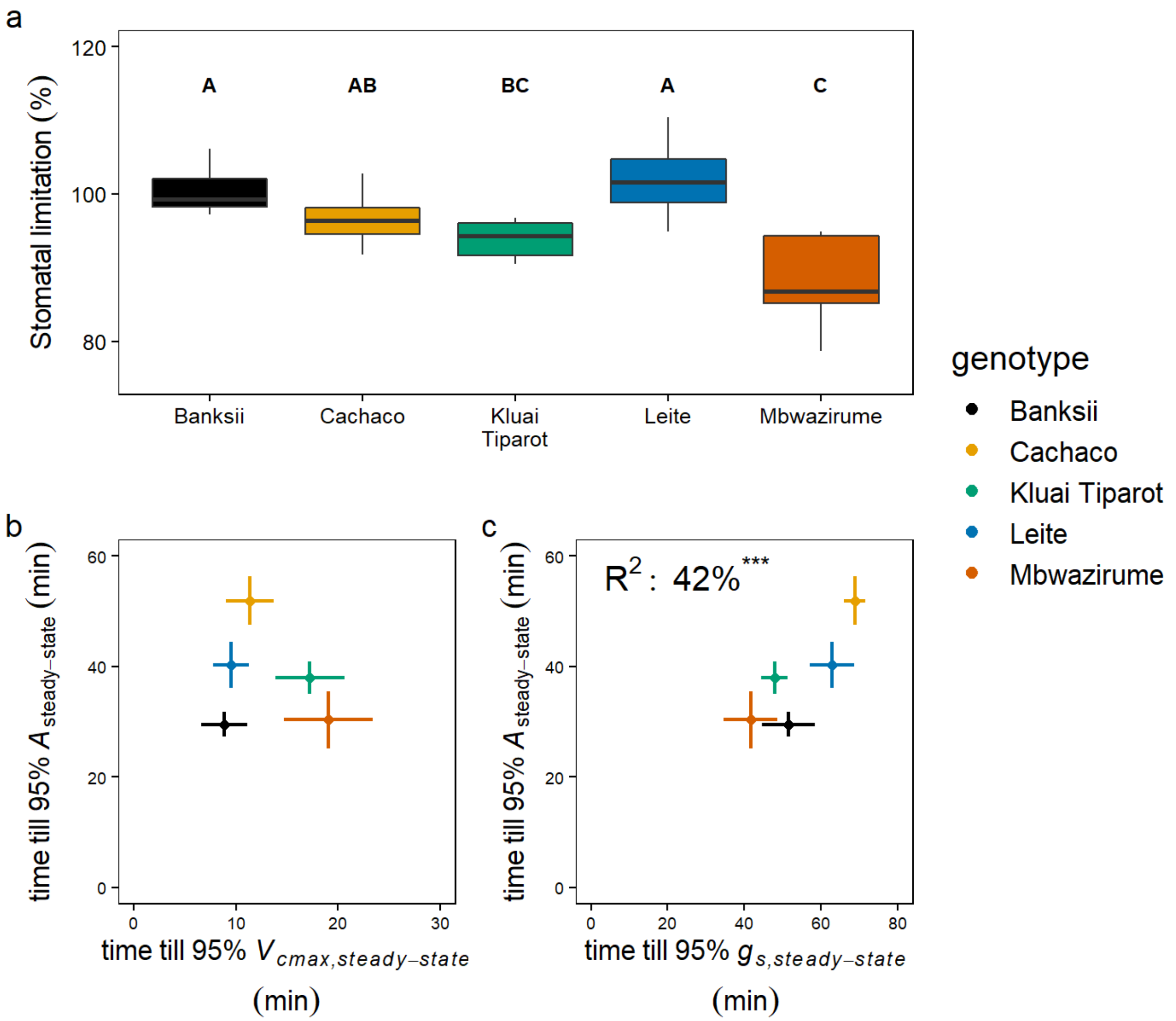

Supplemental Fig. S3 Stomatal limitation of photosynthesis (*A*) and timings until steady-state values of stomatal conductance (g_s), photosynthesis and maximum velocity of Rubisco for carboxylation (V_{cmax}) were reached after an increase in light intensity from 100 µmol m^{-2} s^{-1} to 1000 µmol m^{-2} s^{-1}. (a) Relative stomatal limitations were higher than 89 % for all five banana genotypes. (b) Time to reach 95% of steady-state *A* was not correlated to the time to reach 95% of steady-state V_{cmax} , (c) but was significantly correlated to the time to reach 95% of steady-state g_s. Different letters indicate significant differences between genotypes (post hoc Tukey HSD test, P < 0.05; A>B>C). The bold middle line in boxplots represents the median. The box is confined by the first and third quartile and the whiskers extend to 1.5 times the interquartile distance. *** represents a significant correlation with P < 0.001 (Pearson's correlation). Points and error bars represent mean ± SE (n = 7-8).

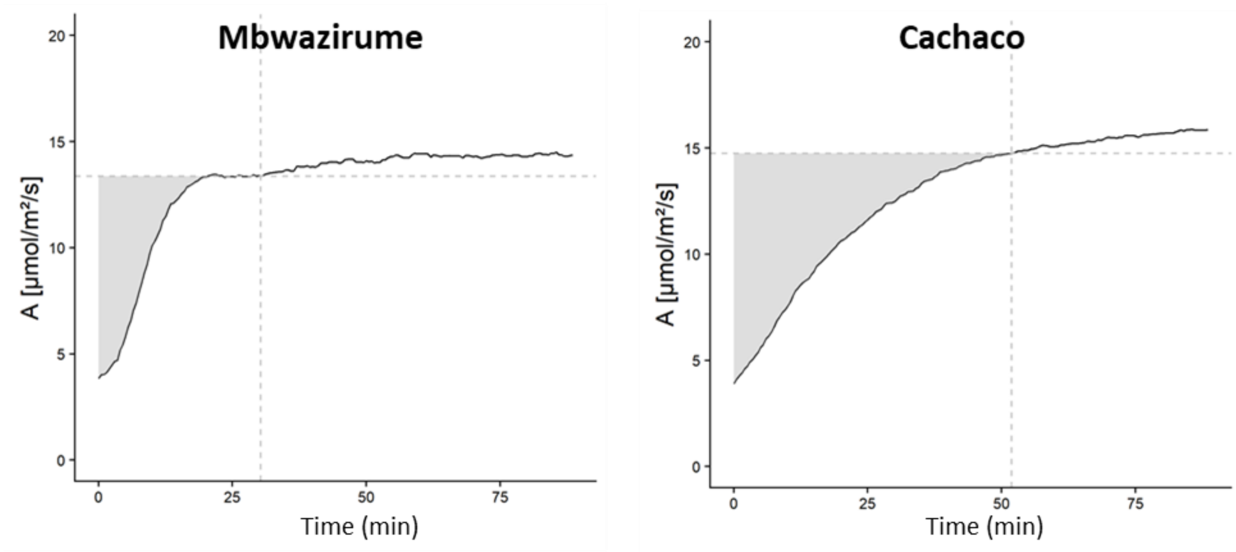

Supplemental Fig. S4 Increase in photosynthesis (*A*) after increasing the light intensity from 100 µmol m^{-2} s^{-1} to 1000 µmol m^{-2} s^{-1}. *A* was considered limited until 95 % of steady-state *A* was reached (dashed line). In grey the percentage limitation of *A* is shown. Data represent the mean kinetic *A* response of the genotypes Mbwazirume and Cachaco (n=7-8).

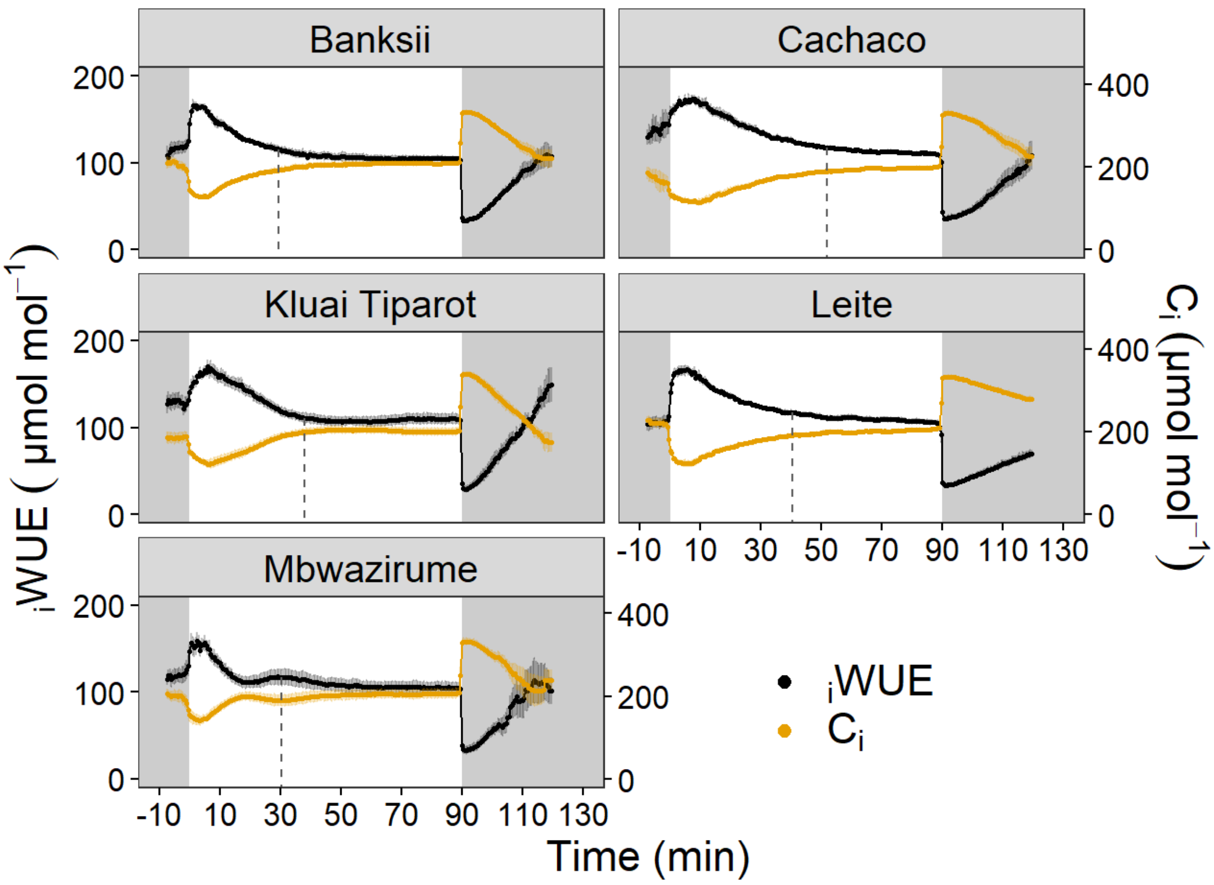

Supplemental Fig. S5 Response of intrinsic water use efficiency ($_i$WUE) and the intracellular CO_2 (C_i) to a step increase and decrease in light intensity from 100 to 1000 µmol m^{-2} s^{-1} and back. Grey and white areas indicate time periods of 100 and 1000 µmol m^{-2} s^{-1}, respectively. Dashed lines indicate when 95 % of steady-state photosynthesis was reached. Grey error bars represent SE (n = 7-8).

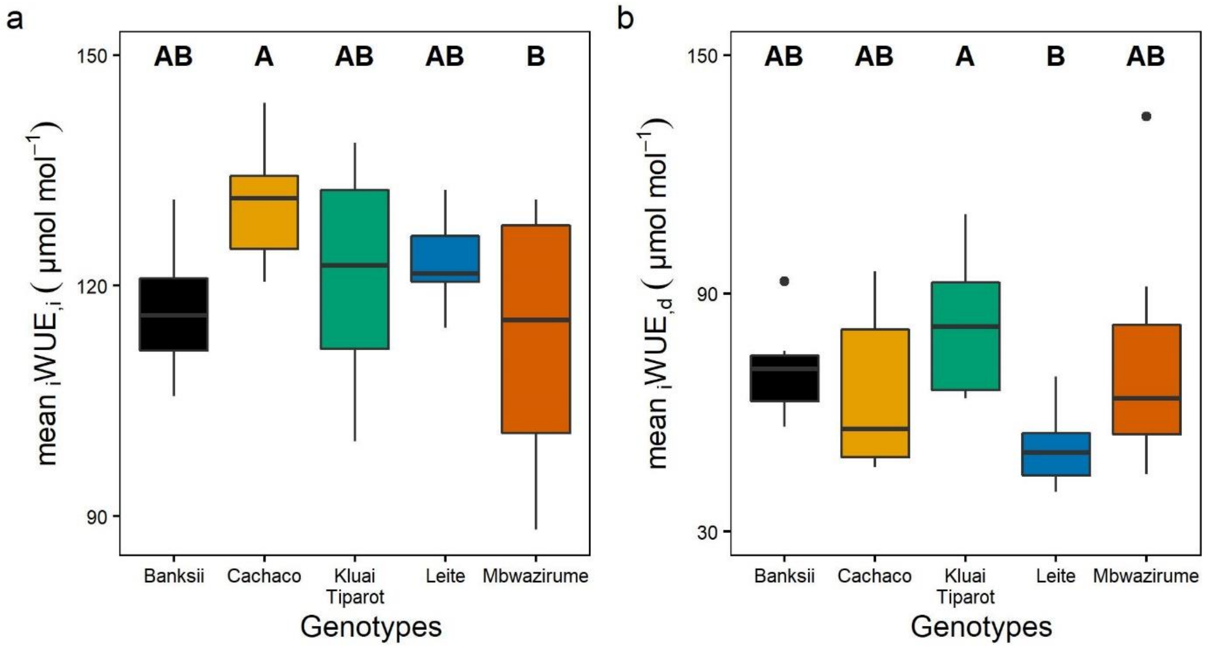

Supplemental Fig. S6 Mean intrinsic water use efficiency ($_i$WUE) after the increase in light intensity from (a) 100 to 1000 µmol m^{-2} s^{-1} and (b) the decrease to 100 µmol m^{-2} s^{-1} afterwards. Different letters indicate significant differences between genotypes (post hoc Tukey HSD test, $P < 0.05$; A>B; n = 7-8). The bold middle line in boxplots represents the median. The box is confined by the first and third quartile and the whiskers extend to 1.5 times the interquartile distance.

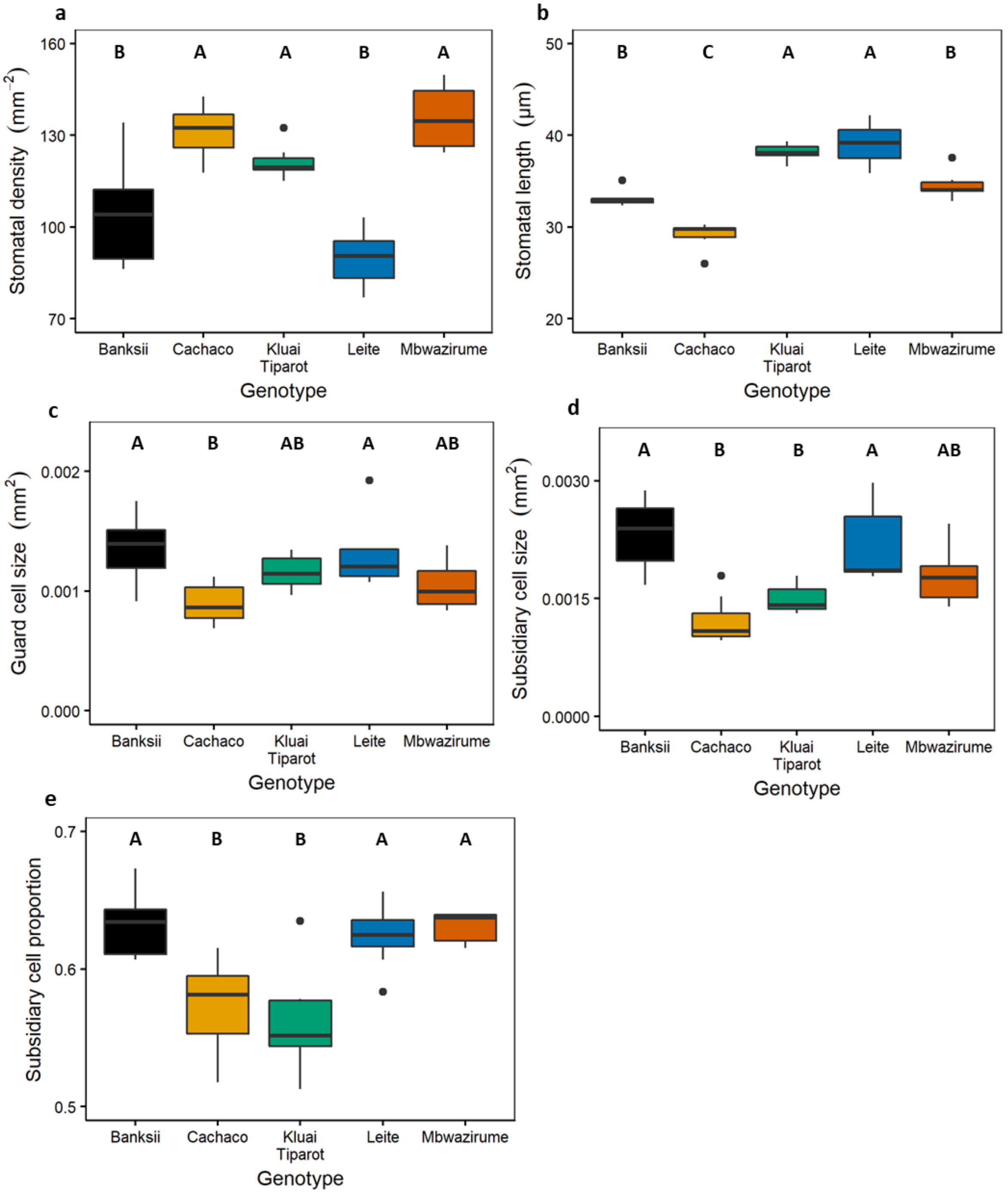

Supplemental Fig. S7 Stomatal density (a), stomatal length (b), guard cell size (c), subsidiary cell size (d) and proportion of subsidiary cells (e) of the five banana genotypes. Different letters indicate significant differences between genotypes (post hoc Tukey HSD test, $P < 0.05$; $n = 6$-8; A>B>C). The bold middle line in boxplots represents the median. The box is confined by the first and third quartile and the whiskers extend to 1.5 times the interquartile distance.

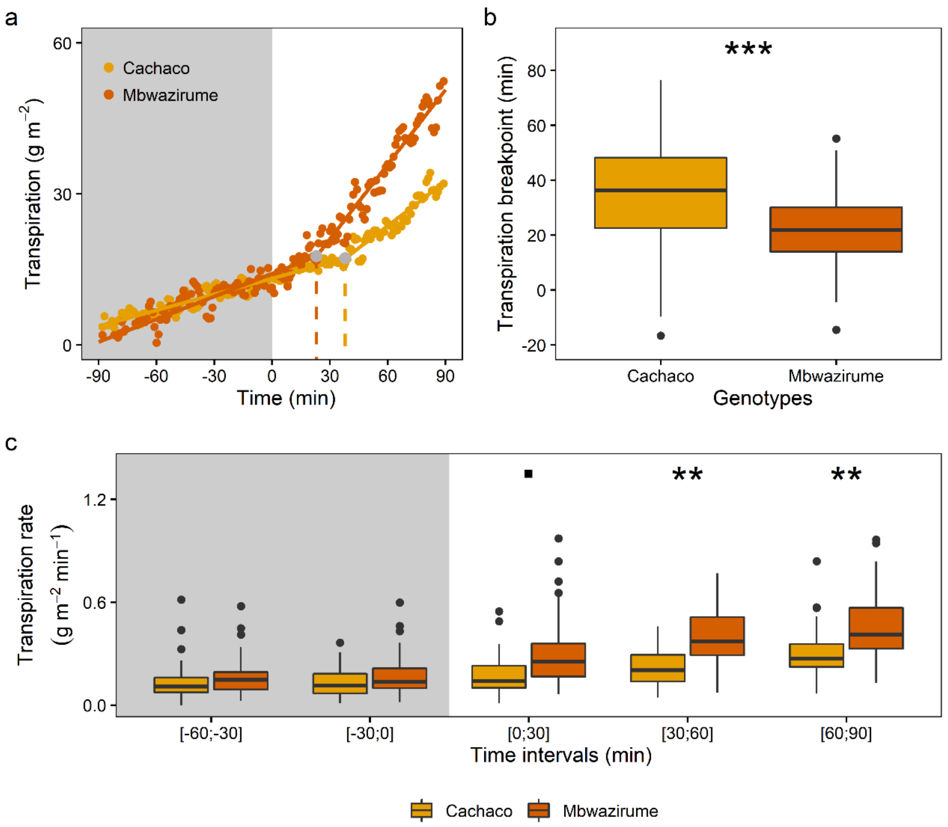

Supplemental Fig. S8 : Gravimetric transpiration rate analysis of genotypes Cachaco and Mbwazirume under gradual increasing light intensity. (a) A breakpoint was identified in whole-plant transpiration after the onset of light in the morning. The transpiration profiles and breakpoints of a representative plant of each genotype on a selected day are shown. (b) The timing of the breakpoint in transpiration after dawn differed significantly between the genotype Cachaco and Mbwazirume ($P < 0.001$, linear mixed-effects model with plant-specific and date-specific random effect). Only data with significant segmented regression ($P < 0.05$) and positive slopes were maintained (87 data points for Cachaco and 96 for Mbwazirume). (c) Transpiration rate after dawn increased faster in Mbwazirume compared to Cachaco. Before dawn transpiration rates did not differ significantly, while within 30 min after dawn, transpiration rate differed significantly (76 datapoints per per time range for Cachaco and 79 for Mbwazirume). Grey areas indicate the time before dawn. (‘.’ for $P < 0.1$, * for $P < 0.05$, ** for $P < 0.01$, *** for $P < 0.001$, linear mixed-effects model with plant-specific and date-specific random effect). The bold middle line in boxplots represents the median The box is confined by the first and third quartile and the whiskers extend to 1.5 times the interquartile distance. Points falling outside the whiskers are considered outliers and plotted as dots.

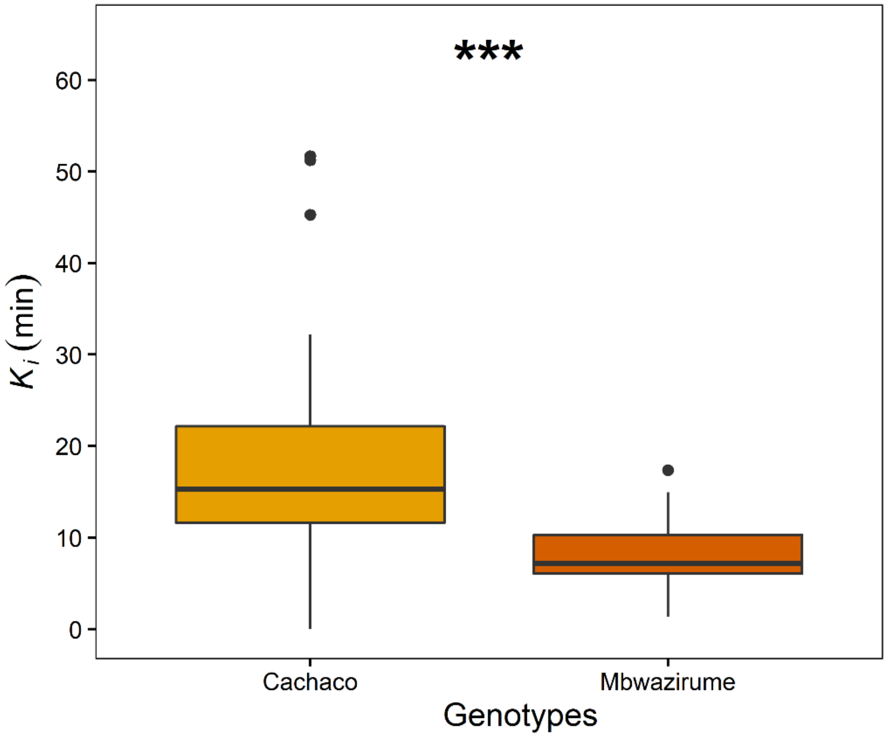

Supplemental Fig. S9 : Modelled time constant (K_i) for the whole-plant transpiration rate increase of genotypes Cachaco and Mbwazirume after a step increase in light intensity from 0 to 120 µmol m^{-2} s^{-1}. (*** for P <0.001, n = 24, linear mixed-effects model with plant-specific and date-specific random effect). The bold middle line in boxplots represents the median. The box is confined by the first and third quartile and the whiskers extend to 1.5 times the interquartile distance. Points falling outside the whiskers are considered outliers and plotted as dots.

Supplemental Table S1 Modelled steady-state and light-induced variables of the stomatal conductance (g_s) response to a step increase and decrease in light intensity from 100 to 1000 µmol m^{-2} s^{-1} for five different banana genotypes.

Genotype	$g_{s,100}$ (mol m^{-2} s^{-1})	$g_{s,1000}$ (mol m^{-2} s^{-1})	K_i (min)	K_d (min)	$Sl_{max,i}$ (mmol m^{-2} s^{-2})	$Sl_{max,d}$ (mmol m^{-2} s^{-2})	λ_i (min)	λ_d (min)
**Banksii**	0.037 ± 0.003^{A}	0.15 ± 0.01^{A}	9.7 ± 0.8^{BC}	6.1 ± 0.6^{BC}	0.074 ± 0.008^{B}	0.132 ± 0.023^{BC}	1.8 ± 1.1^{A}	1.2 ± 0.5^{AB}
**Cachaco**	0.023 ± 0.004^{A}	0.14 ± 0.01^{A}	17.0 ± 1.3^{A}	9.5 ± 1.3^{A}	0.045 ± 0.004^{B}	0.094 ± 0.013^{BC}	2.2 ± 1.4^{A}	2.5 ± 0.9^{AB}
**Kluai Tiparot**	0.030 ± 0.007^{A}	0.16 ± 0.01^{A}	9.7 ± 1.1^{BC}	4.9 ± 0.7^{C}	0.085 ± 0.010^{AB}	0.227 ± 0.032^{A}	6.4 ± 2.4^{A}	2.7 ± 0.2^{A}
**Leite**	0.040 ± 0.003^{A}	0.15 ± 0.01^{A}	13.5 ± 1.6^{AB}	8.6 ± 0.4^{AB}	0.054 ± 0.005^{B}	0.076 ±0.004^{C}	1.5 ± 0.7^{A}	0.5 ± 0.3^{B}
**Mbwazirume**	0.025 ± 0.004^{A}	0.14 ± 0.02^{A}	6.4 ± 0.9^{C}	4.4 ± 0.5^{C}	0.120 ± 0.021^{A}	0.167 ± 0.012^{AB}	2.2 ± 1.1^{A}	2.6 ± 0.5^{AB}

$g_{s,1000}$ and $g_{s,100}$ represent steady-state gs at 1000 and 100 µmol m^{-2} s^{-1} respectively. K_i and K_d, time constants of stomatal opening and closing response. $Sl_{max,i}$ and $Sl_{max,d}$, maximum slope of g_s response during opening and closing. λ_i and λ_d initial time lag during opening and closing. The data are means ± SE (n = 7-8). Different letters indicate significant differences between genotypes (post hoc Tukey HSD test, $P < 0.05$; A>B>C).

Supplemental Table S2 Time to reach 95%, 90% and 50% of steady-state photosynthesis (*A*), stomatal conductance (g_s) and V_{cmax} after a step increase in light intensity from 100 to 1000 µmol m^{-2} s^{-1} for five different banana genotypes.

Genotype	Time $A_{95\%}$ (min)	Time $A_{90\%}$ (min)	Time $A_{50\%}$ (min)	Time $g_{s,95\%}$ (min)	Time $g_{s,90\%}$ (min)	Time $g_{s,50\%}$ (min)	Time $V_{cmax,95\%}$ (min)	Time $V_{cmax,90\%}$ (min)
**Banksii**	29.5 ± 2.2^{B}	23.1 ± 2.5^{B}	5.4 ± 1.1^{AB}	51.5 ± 6.9^{AB}	41.9 ± 5.4^{AB}	10.5 ± 1.2^{B}	8.9 ± 2.2^{A}	5.9 ± 1.0^{B}
**Cachaco**	51.9 ± 4.4^{A}	39.8 ± 3.5^{A}	12.5 ± 2.5^{A}	68.8 ± 2.8^{A}	56.6 ± 3.7^{A}	21.1 ± 2.6^{A}	11.4 ± 2.4^{A}	8.6 ± 1.7^{AB}
**Kluai Tiparot**	37.9 ± 2.9^{AB}	31.3 ± 3^{AB}	9.8 ± 2.4^{AB}	47.9 ± 3.4^{AB}	40.2 ± 2.5^{AB}	16.6 ± 3.0^{AB}	17.3 ± 3.4^{A}	9.1 ± 1.3^{AB}
**Leite**	40.3 ± 4.2^{AB}	27.9 ± 2.7^{AB}	4.6 ± 1.0^{B}	62.8 ± 5.8^{AB}	53.0 ± 4.8^{AB}	12.8 ± 1.2^{B}	9.5 ± 1.8^{A}	6.8 ± 1.0^{B}
**Mbwazirume**	30.3 ± 5.2^{B}	19.4 ± 3.2^{B}	7.3 ± 0.8^{AB}	41.6 ± 6.9^{B}	35.9 ± 5.4^{B}	9.4 ± 0.8^{B}	19.1 ± 4.3^{A}	15.1 ± 3.8^{A}

The data are means ± SE (n = 7-8). Time till 50% of Vcmax is not shown as timings often coincided with the step increase in light intensity. Different letters indicate significant differences between genotypes (post hoc Tukey HSD test, $P < 0.05$; A>B).
